# DIGITAL DANGERS: TECHNOLOGY-FACILITATED ABUSE AMONG ADULTS AGE 50+

**DOI:** 10.1093/geroni/igad104.0867

**Published:** 2023-12-21

**Authors:** Alycia Bayne, Elizabeth Mumford, Caroline Lancaster, Jackie Sheridan-Johnson

**Affiliations:** NORC at the University of Chicago, Chicago, Illinois, United States; NORC at the University of Chicago, Chicago, Illinois, United States; NORC at the University of Chicago, Chicago, Illinois, United States; NORC at the University of Chicago, Chicago, Illinois, United States

## Abstract

Older adults (age 50+ years) are adopting new technologies to increase their independence, prevent social isolation, and help them to age in place. However, digitalization introduces new risks for technology-facilitated abuse (TFA). TFA is any form of online abuse including threats, identity theft, financial fraud, among other forms of victimization. Limited research has explored TFA in general population samples among older adults in the U.S. With support from the U.S. Department of Justice, NORC at the University of Chicago conducted a survey of TFA experiences in a nationally representative sample of n=1,011 U.S. adults aged 50 years and older. Latent class analyses were applied to understand the pattern of older adults’ exposure to ten different forms of TFA, resulting in three classes distinguished by the number of different forms of TFA experienced. This session describes findings from the study, such as socio-economic characteristics associated with TFA profiles, the respondents’ relationship to the perpetrator, behaviors taken in response to TFA, and resulting harms. Among key findings are that six in 10 community-dwelling adults age 50+ have experienced at least one form of TFA in their lifetime. The most common forms of TFA experienced were financial abuses and identity theft. Different demographic groups of older adults experienced different victimization patterns. For example, those who identify as LGBQA were at a much higher risk of experiencing TFA. This session concludes with research and education opportunities for preventing TFA across multiple levels among older adults.

